# Intracellular Electric Field and pH Optimize Protein Localization and Movement

**DOI:** 10.1371/journal.pone.0036894

**Published:** 2012-05-18

**Authors:** Jessica Cunningham, Veronica Estrella, Mark Lloyd, Robert Gillies, B. Roy Frieden, Robert Gatenby

**Affiliations:** 1 Department of Radiology, Moffitt Cancer Center, Tampa, Florida, United States of America; 2 Department of Mathematical Oncology, Moffitt Cancer Center, Tampa, Florida, United States of America; 3 Department of Analytic Microscopy, Moffitt Cancer Center, Tampa, Florida, United States of America; 4 College of Optical Sciences, University of Arizona, Tucson, Arizona, United States of America; Semmelweis University, Hungary

## Abstract

Mammalian cell function requires timely and accurate transmission of information from the cell membrane (CM) to the nucleus (N). These pathways have been intensively investigated and many critical components and interactions have been identified. However, the physical forces that control movement of these proteins have received scant attention. Thus, transduction pathways are typically presented schematically with little regard to spatial constraints that might affect the underlying dynamics necessary for protein-protein interactions and molecular movement from the CM to the N. We propose messenger protein localization and movements are highly regulated and governed by Coulomb interactions between: 1. A recently discovered, radially directed E-field from the NM into the CM and 2. Net protein charge determined by its isoelectric point, phosphorylation state, and the cytosolic pH. These interactions, which are widely applied in elecrophoresis, provide a previously unknown mechanism for localization of messenger proteins within the cytoplasm as well as rapid shuttling between the CM and N. Here we show these dynamics optimize the speed, accuracy and efficiency of transduction pathways even allowing measurement of the location and timing of ligand binding at the CM –previously unknown components of intracellular information flow that are, nevertheless, likely necessary for detecting spatial gradients and temporal fluctuations in ligand concentrations within the environment. The model has been applied to the RAF-MEK-ERK pathway and scaffolding protein KSR1 using computer simulations and in-vitro experiments. The computer simulations predicted distinct distributions of phosphorylated and unphosphorylated components of this transduction pathway which were experimentally confirmed in normal breast epithelial cells (HMEC).

## Introduction

**Figure 1:**
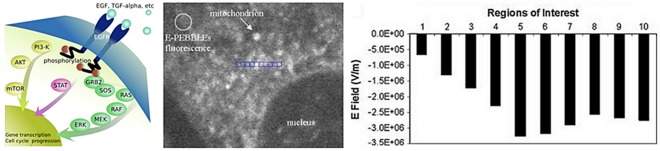
The EGFR pathway and intracellular electric field. (Left) Typical presentation of EGFR pathway. The proteins are not drawn to scale and, as a result, the limitation of random walk in allowing rapid and reliable transmission of information by random walk is underestimated. In fact, the distance from the cell membrane to the nucleus is about 1,000 protein diameters. (Right) Measurements of the intracellular electric field using nano-voltmeter from Tyner et al. [10]. The 10 E-field values in the bar graph on the right are those within the respective 10 blue boxes on the left. Note the decline in the electric field with distance from the nuclear membrane as well as the local perturbation caused by the presence of a mitochondria on regions 5, 6, and 7.

Normal mammalian cell function requires continuous processing of environmental information encoded in ligands that bind to cell membrane (CM) receptors [1]. The molecular pathways that carry (transduce) this information from the CM to the nucleus (N) have been extensively investigated. The components and interactions in these pathways are well-characterized and disruption of one or more of them is almost universally observed in cancer [2]–[4]. Although proteins may be transported via cytoplasmic streaming and microtubular networks, multiple studies have demonstrated messenger proteins move freely in the cytoplasm [5]–[9]. However, in the current cell model, protein communication networks are usually depicted schematically with little consideration of the actual physical motion of the constituent proteins. In the MAPK pathway (see Figure 1), for example, the movement of the messenger proteins is not explicitly integrated into the model but it appears that random motion is sufficient to permit the protein-protein interactions and movement to the N. However, *Figure 1* *is not drawn to scale and significantly underestimates the physical demands of signal transduction.* In fact, signal flow from the CM to NM requires a diffusion distance of about 1,000 protein diameters. Similarly, the probability for collisions between widely dispersed and relatively sparse proteins is not considered. Activated RAS is commonly said to “recruit" RAF to the membrane [2], but this provides no physical mechanism to govern that interaction. RAF is typically present in low concentrations (e.g. 0.013 µM [4] or 8–20,000 molecules/cell). Thus, random interactions between widely dispersed RAF and membrane-bound pRAS would be relatively rare. While scaffolding proteins facilitate interactions, they still must gain proximity through random impacts.

Here we ask if random walk dynamics could account for the molecular collisions necessary to transmit information between pathway components and ultimately carry that information to the nucleus in a timely and efficient manner. As shown below, computer simulations demonstrate that movement by random walk alone significantly limits the speed, accuracy, and efficiency of information transduction.

Our fundamental hypothesis [1], [10] is protein movement and localization is, in fact, highly regulated through Coulombic interaction between the net electric charge of messenger proteins and a recently-measured intracytoplasmic electric (E) field governs protein movement and localization within the cytosol. We propose these dynamical interactions represent a critical but previously unknown component of cellular biology that optimizes signal transduction and information acquisition.

The net molecular charge on each protein or protein complex depends on its isoelectric point (IEP), the cytosol pH and its phosphorylation state (each phosphate adds roughly 2 negative charges). This component of our model is widely used in electrophoresis although it has not been previously applied to cell biology.

The presence of an intracellular E-field has only recently been appreciated [1], [10]. An electrostatic potential across the NM, producing a positive charge on the cytoplasm-facing surface, was first measured in the 1960’s [11]–[13]. However, prior theoretical estimates using the Gouy-Chapman model of E-fields arising from a charged surface predicted a Debye length (roughly the field distance) of 1 nm from the NM [14]. However, the conventional calculation assumes the NM is an impermeable surface. In fact, large pores exist within the NM allowing rapid movement of diffusible ions. This flux of ions through the pores prevents them from screening the NM charge and creates a counter-current as the ions flow from the nucleus into the endoplasmic reticulum where membrane pumps return them to the cytoplasm [15], [16]. Our model predicted a Debye length of 3 to 4 µm, roughly the distance between the NM and CM [1]. Independently, Tyner et al. [10] used nano-voltmeters to demonstrate an intracytoplasmic E-field that quantitatively and qualitatively agreed with model predictions.

Here we present a multidisciplinary study examining the expected localization and movement of messenger proteins as a result of these interactions.

Initial computational models demonstrate that diffusion dynamics alone disperse messenger proteins throughout the cytoplasm prior to entry into the N resulting in complete loss of information regarding the time and location of ligand binding. However, the isoelectric focusing model (IEFM) produces rapid and direct transduction of signal from the membrane receptor allows information regarding the time and location of receptor binding to be conveyed.

We then apply the model to the MAPK pathway. In the presence of an electric field and the measure pH gradient, the simulations predict that RAF (with an isoelectric point [IEP] of about 9.2) is localized to the cytoplasm adjacent to the cell membrane while MEK and ERK (IEP 6.1 and 6.2 respectively) are localized to the cytoplasm closer to the nuclear membrane. Phosphorylation of RAF by RAS in the cell membrane, adds multiple negative charges and the resulting Coulomb interactions with the intracytoplasmic field produce rapid and direct movement of pRAF (transit time less <0.1 sec.) toward the nuclear membrane. Interactions with MEK and ERK which are present in much higher concentration than RAF allows signal amplification. Removal of the phosphate from RAF causes a return to its baseline isoelectric point and rapid relocation back to the cell membrane where it is available for subsequent signal transduction.

The expected steady state distribution of messenger proteins in the MAPK proteins was predicted using a purely diffusion dynamics and the proposed IEFM. Experimental observations in HMEC cells were consistent with the IEFM predictions.

## Materials and Methods

### Mathematical Models

In experimental studies using fluorescent methods, messenger proteins appear to be free diffusible in the cytoplasm [2]–[9]. Thus, a molecular dynamics algorithm is used to simulate the multiple proposed intracellular signaling schemes. A molecular dynamics simulation allows snapshots of moments in time as individual particles progress under physical principles. This facilitates direct visual comparison between the model results and the available microscopy images.

### Computer Simulations

#### Simulating molecular dynamics of signaling pathways

The movement of messenger proteins is modeled using molecular dynamics techniques as a general n-body problem using Brownian diffusion and Coulomb interactions instead of the traditional n-body gravitational interactions. Each messenger protein is modeled as a particle that is assigned a set of initial conditions including position, velocity, acceleration, isoelectric point, and phosphorylation state. Given this information, the mathematical laws that define the physical motion of these particles can be used to predict their future state for any time in the past or future.

To determine these future states after some time Δ*t* all physical influences on motion for each particle must be calculated for a number of discrete time points between the starting time and the desired 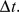 The length of this discrete time chunk, Δ*t*, plays a significant role in simulation design. A smaller 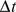 will provide a higher time resolution model, but requires many more iterations and therefore more time and computing power. A Δ*t* is chosen to maximize the time resolution while keeping in the constraints of computing power available. Δ*t* = 0.0001 *sec* was chosen for the simulations shown within this manuscript.

The model assumes both a spherical cell wall and nuclear membrane. The radius of the nucleus is 3 µm and the radius of the cell membrane is 5 µm. This dictates the shortest travel distance from the cell wall to the nuclear membrane is 2 µm, if the particle moves directly towards the cell origin immediately after leaving the cell wall. Two different views of the simulated cell are used in the manuscript. One shows the entirety of the spherical cell with the nucleus in the center. The other view shows a “core sample" of the spherical cell where the nuclear membrane is located at the bottom and the cell wall is located at the top. This second “core sample" view allows for a higher resolution analysis of the dynamics of the simulated particles.

#### Signal transduction via Brownian motion

The molecular dynamics simulation models Brownian motion as a Wiener process, which assumes continuous-time stochastic dynamics. To model this process, four assumptions were met:

1. Movements were made at regular time intervals.
2. Movements of a set length were made defined by the diffusion coefficient.
3. The direction of each step was randomly chosen in 3 dimensions.
4. All particles were independent of each other.

Assumption 1 was met under the formulation of a molecular dynamics model where particles were updated at regular discrete time intervals. Assumption 2 requires the calculation of the diffusion coefficient of a particle in a medium with a low Reynolds number, such as the cytoplasm. The Einstein-Stokes equation below allows for an approximation of the diffusion coefficient to be made for messenger proteins.
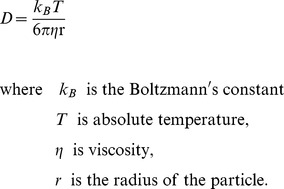



The value of the cytoplasmic diffusion coefficient has been investigated experimentally and in this simulation was defined as 
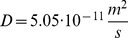
. Theoretical calculations yielded similar coefficient values.

Assumption 3, random movement in three dimensions, was met by creating a direction vector for each messenger protein during each time step using a random number generator. Each particle was updated individually under Brownian motion simply by moving the particle the length defined by the diffusion coefficient in the random direction created.
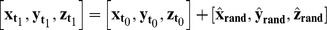



Additional analysis was performed to verify that the diffusion simulated by the discrete molecular dynamics model paralleled well known theoretical mathematical predictions. Using the theory of root mean square (RMS), the diffusion of particles can be described as a distribution of particles over time. This distribution is known as the RMS displacement and is given in three dimensions below.
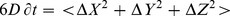
where *D* is the diffusion coefficient, 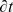 is the elapsed time, and 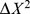 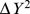 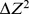 are the average movement in either X, Y, or Z directions respectively.

The theoretical RMS displacement in any direction after a time 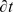 was recreated using the Wiener process simulation. In this way, the model was sufficient to correctly model Brownian motion. The biased movement of these particles caused by the electric field and pH isoelectric focusing is performed separately from this diffusion equation. In this way the bias is not evident in the pure diffusion RMS equation. This is done to allow modeling of the pure diffusion portion of the particles motion separate or together with the electric field and pH effects. (Figure S1).

#### Signal transduction via Coulomb Interactions

The intracellular electric field in this model assumes a radially declining value and is based on both theoretical considerations and experimentally determined values from Tyner et al [10] (Figure 1). We do not ascribe a precise mechanism to the field and simply assume we can follow Gauss’s Law and allow the charged nuclear membrane to exhibit the same attributes as a point charge at the center of the nucleus. In this way, the nucleus can be considered as an individual fixed particle instead of a surface.

As noted above phosphorylation of messenger proteins, in addition to altering their configuration and function, adds negative charges. The molecular dynamics simulation models Coulomb interactions of these charges with the intracellular electric field using the mathematical formulation of the n-body problem. The movement of each particle undergoing Coulomb interactions is found by summing the total force acting upon that particle at each iteration. The forces in our simulation were defined by Coulomb’s law.
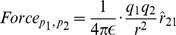
Where 
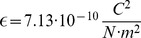
 or 80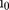, the intracellular electric constant, *q*_1_ and *q*_2_ are the charges of two interacting particles *p*_1_ and *p*_2_ (Coulombs), *r* ^2^ is the distance squared between two interacting particles *p*_1_ and *p*_2_(*m* ^2^), 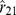 is the unit direction vector of the force.

When calculating the total force on a particular particle, the Coulomb interactions were calculated between that particle and every other particle in the system, including the nucleus, known as full-interaction. These multiple forces are summed to equal the total force on the particle of interest.
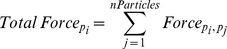
Once the total force on a particle is known, Newton’s laws can be used to translate this force into acceleration, velocity, and ultimately the particles new position.



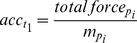


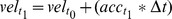
Once the new velocity is calculated it must be modified to obey the intracellular drag forces. The intracellular drag force is calculated using Stokes’ law for particles with low Reynolds numbers, 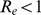. This approximates the linear drag for particles moving through a fluid at relatively slow speeds where there is no turbulence. The equation for this viscous resistance is



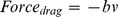
Where *b* is the drag constant, *v* is the velocity of the particle 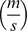.

The drag constant *b* depends on the properties of the fluid and the dimension of the particle and is defined for low Reynolds number particles below.
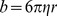
where 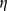 is the fluid viscosity. For the intracellular environment 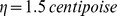, *r* is the Stokes radius of the particle. For this model 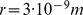, This gives an intracellular drag constant 
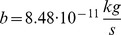
.

This allows for the calculation of the theoretical terminal velocity of a particle under its total Coulomb force. The terminal velocity will increase as the Coulomb force increases. The terminal velocity is reached when the Coulomb force equals the drag force.
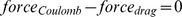



Solving this gives the terminal velocity of a particle *p_i_* under a specific total Coulomb force as
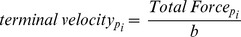
If the velocity calculated using Newton’s second law directly from the total Coulomb force is greater than this terminal velocity, the velocity of capped at the terminal velocity. Any velocity below the terminal velocity is accepted.



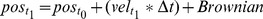
where 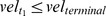.

All particles that undergo Coulomb interactions also undergo Brownian motion. Therefore, once the new position is found due to the Coulomb forces, the position is also updated as described by Brownian motion above. In this way the messenger proteins undergoing Coulomb interactions experience: 1. Attractive Coulomb forces towards the positively charged nucleus, 2. Repulsive Coulomb forces away from every other phosphorylated particle, and 3. Brownian motion.

#### Isoelectric focusing of intracytoplasmic proteins

Each particle has a specified isoelectric point (IEP) that is defined as the environmental pH value at which the particle has zero net charge. If a particle is in a lower pH than its IEP, it has an increasing probability (with decreasing pH) of becoming protonated and carrying a net positive charge. If the particle is in a higher pH than its IEP, it has an increasing probability (with increasing pH) of becoming deprotonated and carrying a net negative charge. These probabilities were modeled using speciation diagrams.

Before the Coulomb interactions are modeled, the charge of each particle is calculated based on its position within the pH gradient and phosphorylation state. Using the equations for the speciation diagram specific to the particles IEP, the particle can be assigned a new charge value based on probability. Consider using a particle with a IEP  = 6.7, that is now in an environment where the pH  = 6.5. (Figure S2) This corresponds to a 10% chance of becoming protonated and a 90% chance of remaining neutral. Using a random number generated from a uniform distribution [0, 1] the new charge of the particle is stochastically determined.

This process can be completed for any IEP in any environmental pH. These changes in net charge will cause the particles to undergo different Coulomb interactions based on where they are located within the cytoplasm at each iteration.

The negative charge associated with phosphorylation is also affected by the pH value. A phosphate molecule within the pH of the cytoplasm has a 50% chance of carrying one negative charge and a 50% chance of carrying two negative charges. Combining this phosphorylation charge value with the charge value obtained from the protein’s isoelectric point, the total messenger protein charge is obtained.

#### Isoelectric focusing of intracytoplasmic proteins

To model isoelectric focusing, a theoretical pH gradient was added to the cytoplasm. In initial experiments we quantified intracellular pH and documented predictable regional variations. We found the intracytoplasmic pH ranged from 7.2 to 7.4. The perinuclear cytoplasmic pH and the pH adjacent to the cell membrane were relatively acidic when compared to the cytoplasm between these regions (not shown). The values are consistent with reported values although we are not aware that variations in intracytoplasmic pH have been systematically studied.

### Experimental Models

#### Immunofluorescent staining

Cells are then washed with 1X PBS and fixed in 4% paraformaldehyde for 10 minutes, permeabilized in 0.1% Triton for 10 minutes, and blocked in 1% BSA/1X PBS for 30 minutes. After washing cells in 1X PBS, they were serially incubated in primary antibodies, phosphorylated RAF (ab1095) 1∶500 and total RAF (ab18761) 1∶200; phosphyrylated MEK (ab32088) 1∶200 and total MEK (610121 BD) 1∶100; Phosphorylated ERK (ab50011) 1∶200 and total ERK (ab17942) 1∶100. Mouse antibodies were conjugated to a secondary Alexa 488 antibody 1∶500 and Rabbit antibodies were conjugated to a secondary Alexa 647 antibody 1∶500.

#### Mitochondria imaging

HMEC cells were seeded in 35 mm glass bottom plates at 0.05×10^6^ cells/ml. They were incubated at 37°C overnight. After 24 hrs cells were incubated with 0.5 µg/ml Mito Tracker Green for 15 minutes and washed with 1X PBS. Media was replaced prior to imaging.

#### Microscopy

Micrographs of HMEC cells in glass bottom plates or micropattern chips were taken with a Leica TCS SP5 AOBS laser scanning confocal microscope through a 63X/1.40NA Plan Apochromat oil immersion objective lens (Leica Microsystems, Germany). A 405 nm Diode laser line were applied to excite Hoechst nuclear dyes, an Argon 488 nm laser was used to excite SNARF and Alexa 488 antibodies, and a diode 633 nm laser was used to excite Alexa 647. AOBS filters were set to optimally capture emission spectra for each dye. The fluorochrome selection and tunable emissions were used to minimize crosstalk between fluorochromes. Image z-sections for samples were captured with photomultiplier detectors and prepared with the LAS AF software version 2.1.0 (Leica Microsystems, Germany). A 2–3x optical zoom was applied to increase the total magnification.

#### pH image acquisition and analysis

Intracellular pH was measured using 1 µM carboxy SNARF-1 AM acetate in HMEC cells grown on CYTOO micropatterns. After 30 minute incubation with SNARF-1, samples are washed with 1XPBS and media is reapplied. Images are acquired with a Leica TCS SP5 AOBS laser scanning confocal microscope using the Argon 488 nm laser excitation and capturing the dual fluorescence emission of the dye with distinct PMT detectors (560–600 nm; and 620–600 nm), which exhibits a pH dependent spectral shift. A ratiometric analysis of the fluorescence intensities allows us to accurately determine pH based on calibration data. pH values were obtained through ratiometric analysis using Definiens software to determine the pH of 250×250p×l superpixels (arithmetic mean). Regional values were selected.

#### Cell culture

HMEC, normal human mammary epithelial cells, were obtained from Invitrogen Life Technologies Corporation, Carisbad, CA. HMEC cells were maintained as adherent cultures in HuMEC Basal Serum Free Medium (Invitrogen Life Technologies Corporation, Carisbad, CA) supplemented with HuMEC Supplement and Bovine Pituitary Extract (Invitrogen Life Technologies Corporation, Carisbad, CA). Cultures were maintained in standard incubation conditions 37°C and 5% CO_2_.

#### Sample preparation

A significant limitation of measuring spatial distribution of intracellular proteins in conventional cell culture is the variations in cell shape and other morphologic features. To allow experiments on cells of the same shape and morphology, HMEC cells were seeded on 20×20 mm^2^ chips (CYTOO, Framingham, Massachusetts) stamped with a medium (1100 µm^2^) fibronectin micropattern size. CYTOO chips are 170 µm thick coverslips that are manufactured to express fibronectin micropatterns on an organized grid. Individual cells bind to these pre-formed fibronectin micropatterns and consequently each cell adopts the same shape.

## Results

### Signal Transduction Simulations

#### Signal transduction using only random walk

**Figure 2:**
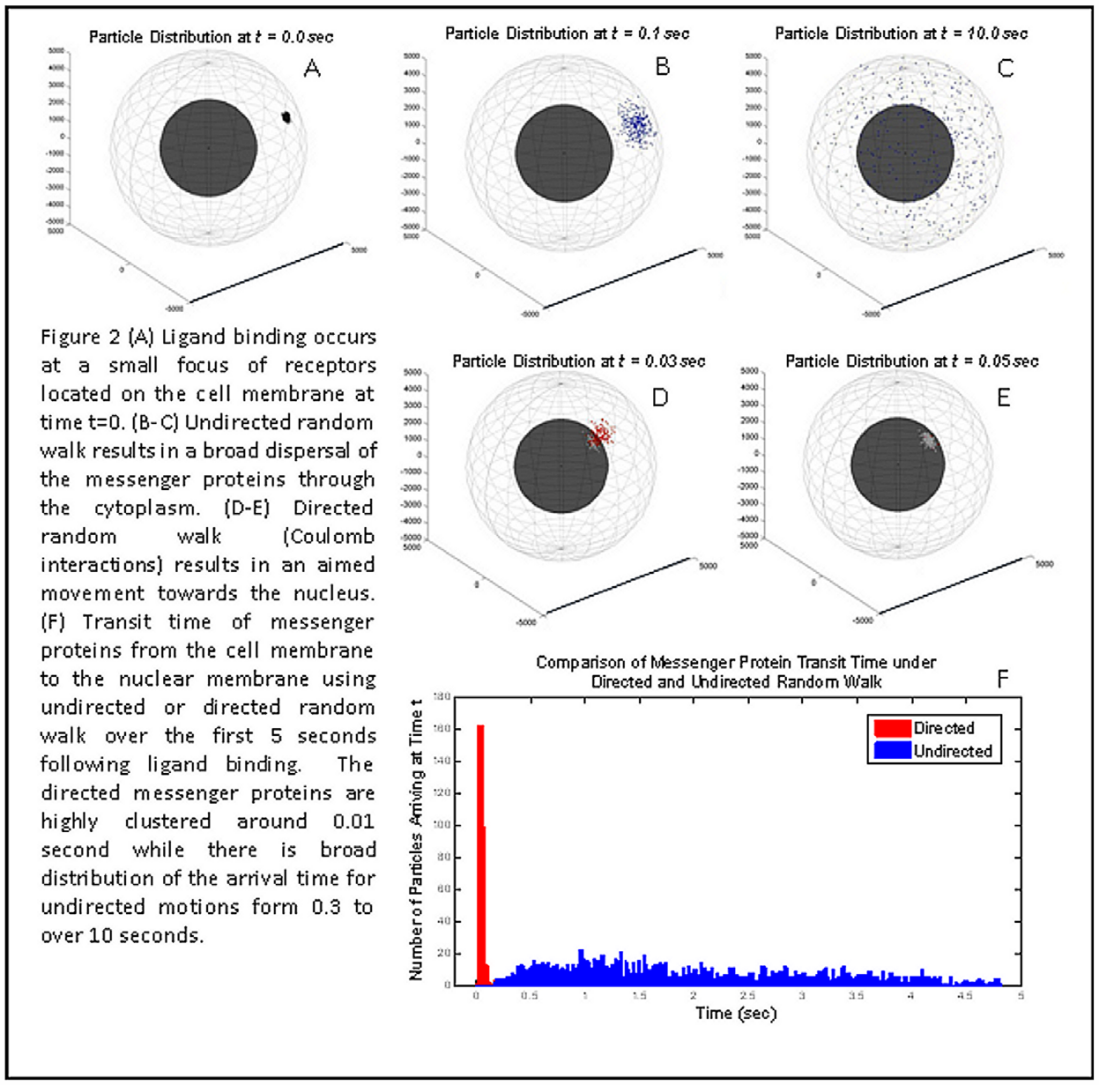
Diffusion of messenger proteins resulting from ligand binding to a small focus of receptors on the cell membrane at time t = 0. By random walk (top panels), there is broad dispersal of the messenger proteins through the cytoplasm. As a result, all information regarding the time and location of ligand binding is lost. By biased random walk (lower panels) due to Coulomb interactions of phophorylated, negatively charged messenger proteins with an intracytoplasmic electric field, the spatial location of ligand binding on the CM is projected onto the NM and the transition time is less than 0.1 second.

Figure 2 shows the movements of a single class of messenger proteins traveling from the CM to the N by Brownian random walk alone versus messenger proteins directed by Coulomb interactions. This model is focused only on protein movement as a result of Coulomb interactions and does not take into account protein IEP or cytoplasmic pH.

At time t  = 0 we assume 100 messenger proteins of each type are phosphorylated on the inner surface of the cellular membrane. This modeled the simultaneous activation of several receptors clustered in one location of the cell membrane. Each phosphorylated protein subsequently travels by either only Brownian random walk or by Coulomb-directed Brownian random walk confined only by the CM and NM. If a particle arrives at the NM it is fixed at its collision location. The time at which the particle arrived at the NM is tallied and the compilation of these transit times is also shown in Figure 2.

The messenger proteins traveling simply by Brownian motion are shown diffusing through the cytoplasm for a total of 10.0 seconds. At the end of these 10 seconds, the majority of the phosphorylated messenger proteins have not arrived at the nuclear membrane. Furthermore, during their transit messenger protein traveling by Brownian motion disperse throughout the cytoplasm so that they may arrive at the nucleus anywhere on the surface of the nuclear membrane, even on the opposite side from the origination point of the protein. While the overall distribution of arrival locations on the nuclear membrane may favor the direction of the origination point, the ratio of signal (proteins that arrived at the projected origination location onto the nucleus) to noise (the proteins that arrive anywhere else on the nuclear membrane) of this information is very low. Thus, information on the location and arrival time for ligand binding at the cell membrane is degraded by random walk.

#### Signal transduction with coulomb forces

**Figure 3:**
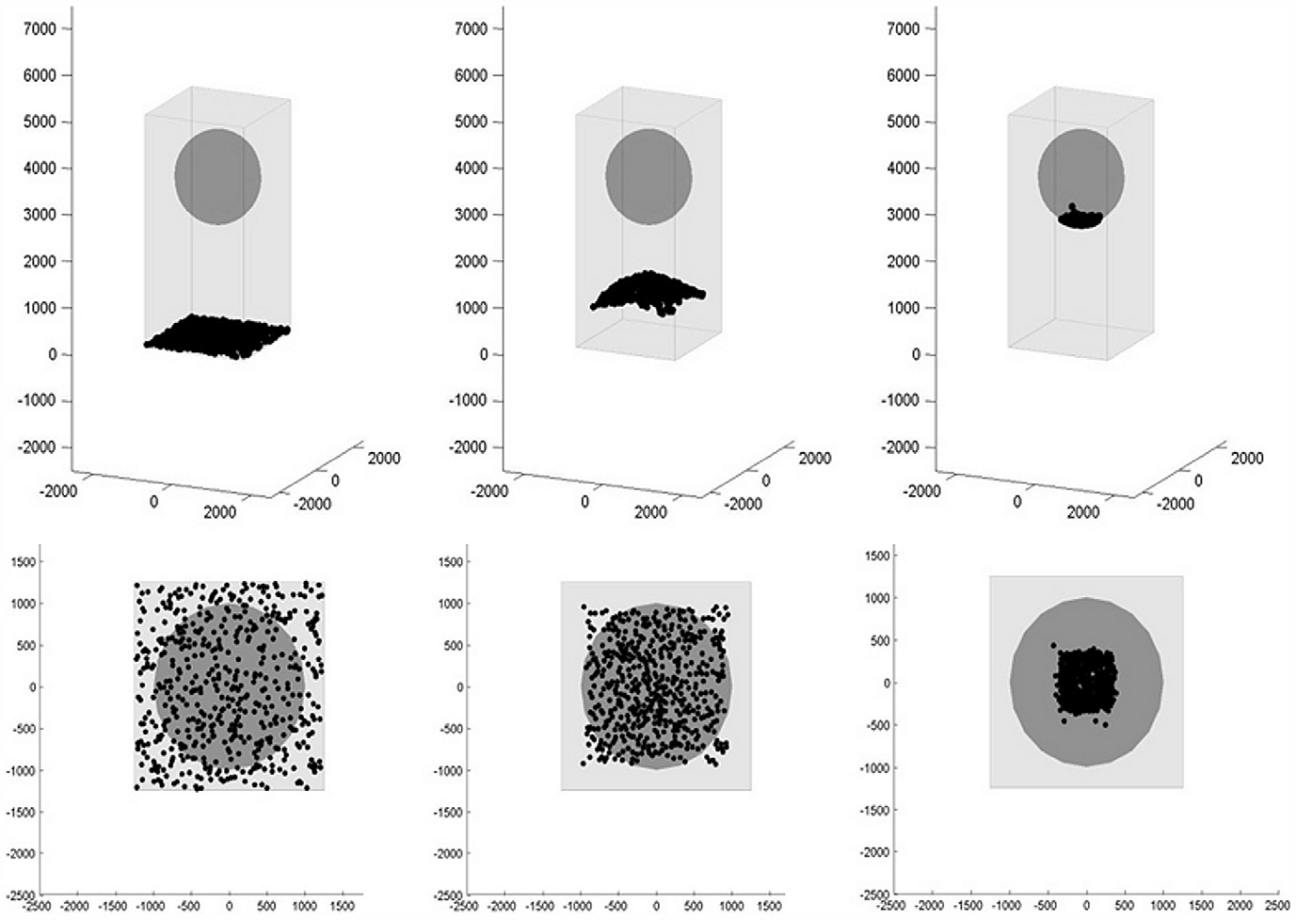
Spatial resolution of ligand binding on cell membrane. Here we assume a cubicle cellular configuration to match the shape frequently seen on epithelial surfaces. We assume that the lower part of the cell is attached to a basement membrane and that ligand binding occurs uniformly and simultaneousl but only in the cell wall attached to the basement membrane (left images). The messenger proteins travel as a wave from the CM to the NM (middle images). The messenger proteins, due to directed motion, can project spatial information on the site of ligand binding onto the NM. This is evident in the recapitulation of the ligand binding pattern in the CM onto the NM in the right images.

Simulations of messenger protein movement via biased random walk due to Coulomb interactions with an intracellular electric field are shown in Figure 2 and 3. All of the proteins arrived at the NM in less than 0.05 seconds (mean of 0.01 sec). In addition, the location on the cell membrane at which the ligand arrived was maintained through the direct protein movement. As seen in Fig 3, the origination location of all of the phosphorylated proteins is projected on the surface of the nuclear membrane. To show this more explicitly, a number of messenger proteins were phosphorylated on a square section of the cell wall and allowed to undergo Coulomb directed movement (Figure 3). Once all of the proteins have reached the nucleus, it can be seen that the original square shape is projected onto the nuclear surface. The nucleus could then readily use this spatial element to gain information about the surrounding environment.

### Application to the MAPK Pathway

#### Modeling MAPK pathway proteins and movement

We simulated the placement and movement of proteins in MAPK pathway assuming an initial resting state and then an abrupt arrival of multiple ligands at the CM. We focus on movement and interactions of the 3 proteins that carry the signal from the CM to the N following ligand binding to epidermal growth factor receptor (EGFR): RAF, MEK, and ERK (Figure 1) [3], [4], [15]. In reality, of course, each protein has multiple isoforms and the reported interactions among the pathway components are variable and complex. In the following model we simplify the pathway in an attempt to define first principles acknowledging that the model is not comprehensive.

**Figure 4:**
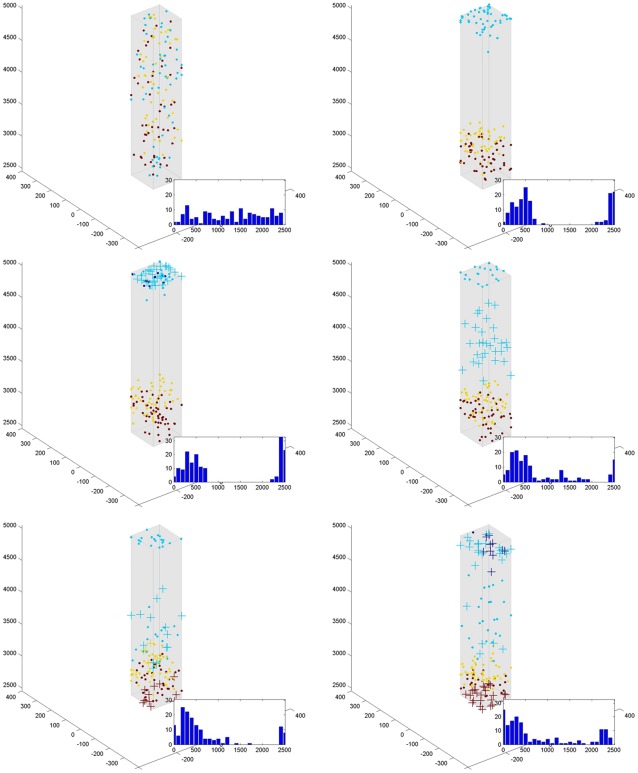
Dynamic modeling of RAF, MEK and ERK after ligand binding. In the initial state, RAF is clustered around the CM and MEK and ERK around the NM. When ligand binds the membrane receptor, RAF is phosphorylated. The negative chareges interact with the intracellular field resulting in rapid (<0.01 sec) movement of pRAF toward the NM. As it reaches the perinuclear region, pRAF encounters and phosphorylates several MEK proteins which, in turn, phosphorylate several ERK proteins. The rapid, direct movement of pRAF provides spatial and temporal information while the interactions with MEK and ERK amplify the signal at the NM. The pRAF is assumed to encounter a phosphorylase after about 30 seconds. The loss of negative charges causes RAF to return to it original isoelectric point with very rapid (<0.01 sec) return to the CM where it is again available for signal transduction.

In the simulation we use an electric field as predicted and measured in prior studies [1], [12] and a pH distribution of 7.2 to 7.4. Simulation of the transduction cascade assumed that 100 ligands bound to EGFR receptors on the cell membrane in the modeling domain at time t = 0. We assume that RAF protein is then activated by association with GTP-RAS, and is subsequently phosphorylated for a period of 30 seconds. After that period, phosphatases act on pRAF, removing the phosphates. When pRAF encounters MEK it results in MEK phosphorylation. Similarly, when pMEK encounters ERK it is phosphorylated. For both MEK and ERK, the region of relatively acidic perinuclear corresponded to the distribution of and we infer that it is a consequence of metabolic H^+^ (CO_2_) production. The phosphates are assumed to be removed by phosphatases after 30 seconds. These protein dynamics are shown in Figure 4.

In the absence of any ligand binding, the MAPK proteins distribution is largely dependent on their IEP. RAF with an IEP of 9.2 is highly concentrated around the CM while MEK and ERK (IEPs of 6.1 and 6.2 respectively) cluster near the NM.

After phosphorylation, pRAF moves rapidly (about 0.1 second) and directly from the CM the perinuclear cytoplasm where it encounters multiple MEK proteins which in turn multiple ERK proteins which then strike the nuclear membrane. Interestingly, the rapid and direct movement of pRAF allows both the time and location of ligand binding to be conveyed to the nucleus. However, interactions with MEK and ERK allow signal amplification without loss of positional information. Finally, we demonstrate that upon loss of phosphate groups RAF moves back to the CM in less than 1 second where it is now ready to transduce the signals from subsequent ligand binding events.

The predicted rapid movement of the MAPK proteins could not be experimentally observed. For this reason, we examined the computation models assuming a steady state with continuous presence of ligand at the CM as would be expected under normal culture conditions. To better capture the full biological dynamics, we added interactions with the scaffolding protein KSR1. Scaffold proteins have been found to play a large role in modulating the signaling strength and regulating the signal amplitude and duration of the MAPK pathway. The overall role of these scaffold proteins is currently under investigation. KSR1 is one of several such proteins that mediate MAPK protein movements but it is well described and its addition to the model seemed reasonable [16]–[18]. For our simulations, we assumed that MEK was usually bound to KSR1 and that this complex interacts with pRAF and ERK permitting the sequence of phosphorylations that result in formation of pERK which then unbinds from KSR1 and moves toward the NM. We also assumed that pERK can bind at a separate site on KSR1 preventing additional pRAF binding.

We modeled these interactions under two scenarios. First, we assumed that no intracellular field was present and examined the expected distribution of proteins with movements governed purely by random walk. Second, we assumed the presence of an intracellular electric field and intracellular pH gradient, with protein localization and movement governed by these physical properties interacting with proteins based on their size and isoelectric point. The cytoplasmic pH was assumed to range from 7.2 near the nucleus to 7.4 in the peripheral cytoplasm based on experimental measurements (not shown). These values are consistent with published reports [19]. In each case, we simulated the expected location of unphosphorylated RAF, MEK and ERK.

**Figure 5:**
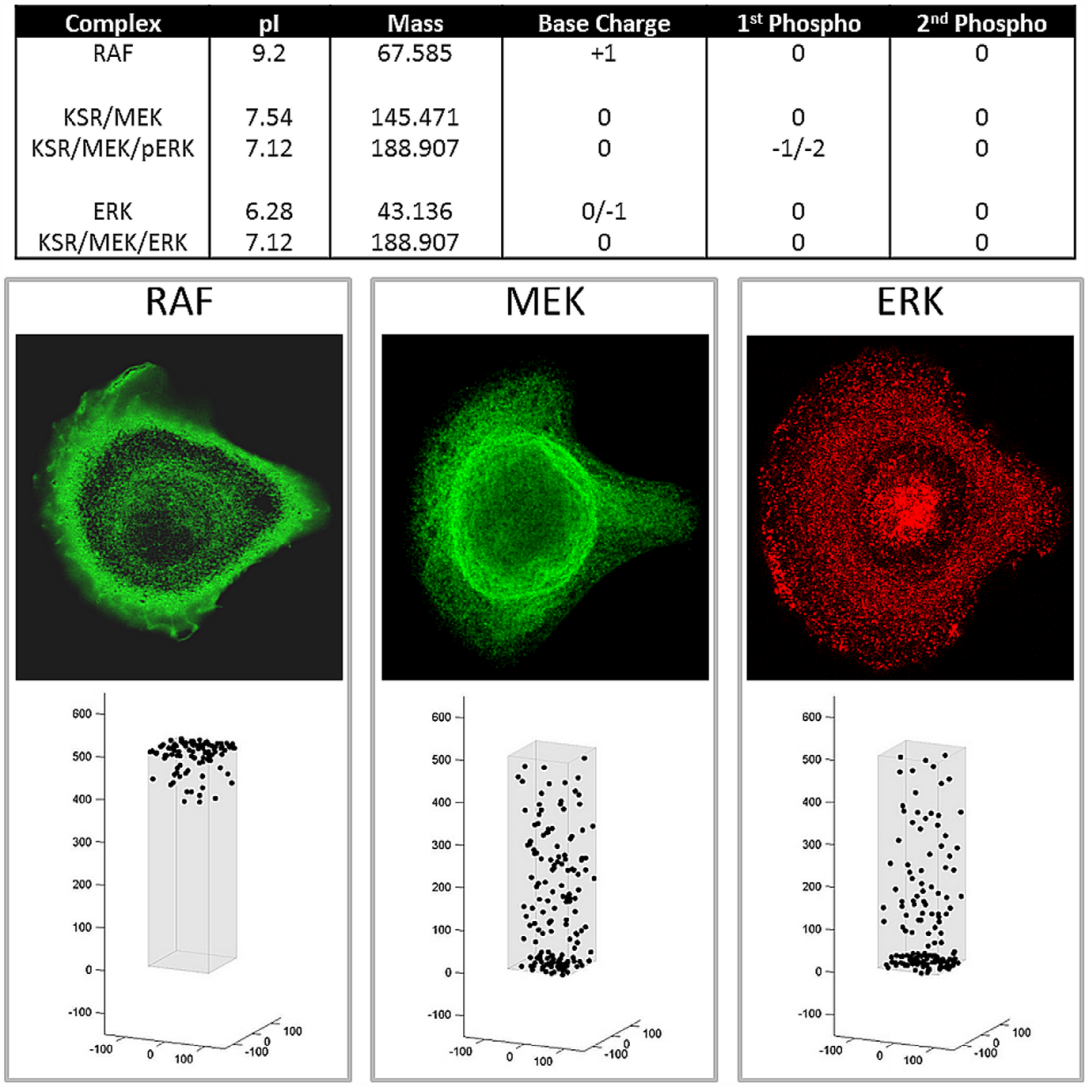
Simulations of steady state distribution of MAPK proteins in culture conditions with continuous presence of EGF. The model assumes the presence of scaffolding proteins KSR1 as outlined in the text. Top panel represents the physical characteristics of RAF, MEK and ERK both free and bound to KSR1 used in the computer simulations. Lower panels represent predicted steady state distribution of RAF, MEK, and ERK in normal cells assuming continuous presence of ligand at the cell membrane and assuming the presence of scaffolding protein KSR1. Middle panels are actual distribution observed in HMEK cells in culture using CYTOO chips so that every cell maintains roughly the same shape.

**Figure 6:**
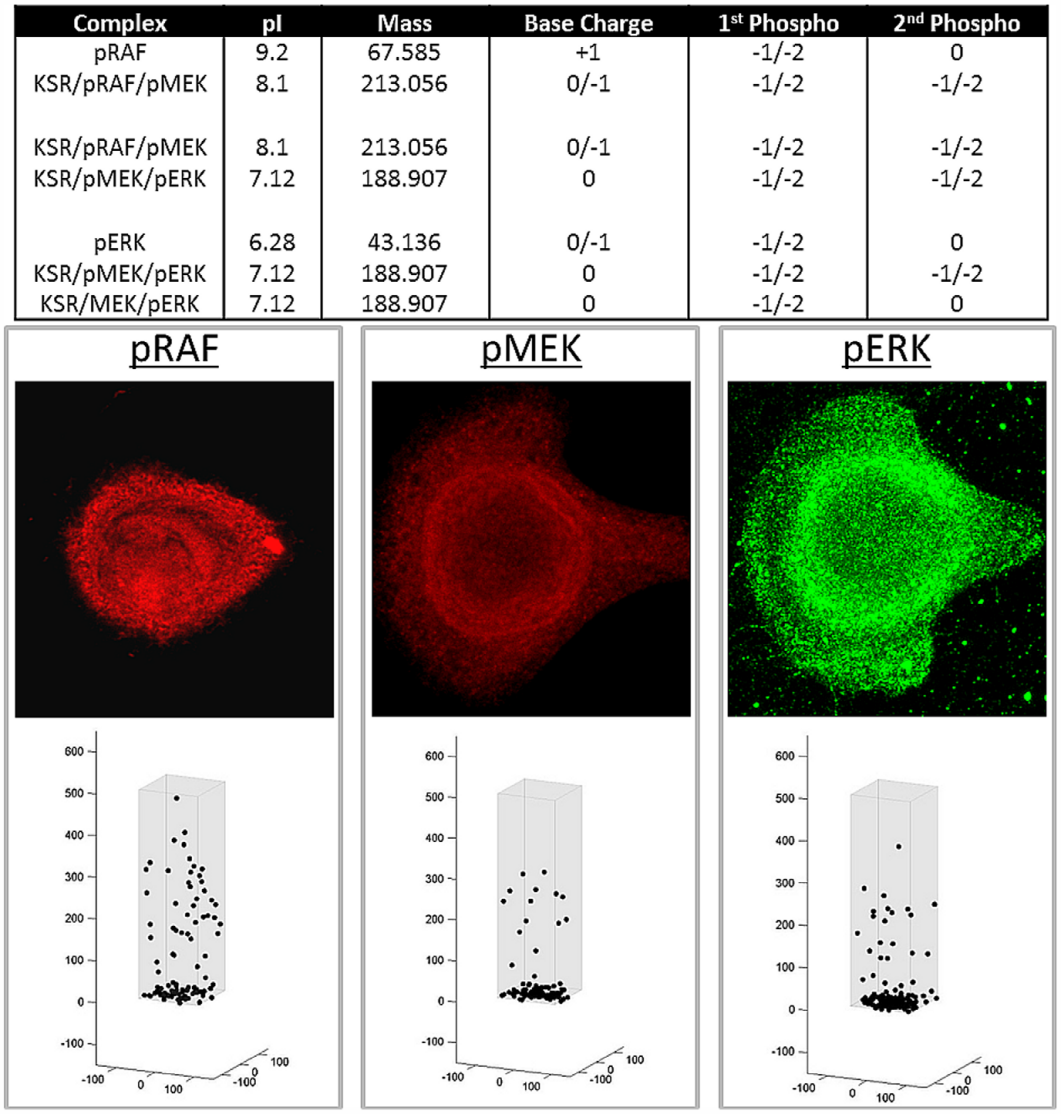
Simulations of steady state distribution of phosphorylated MAPK proteins in culture conditions with continuous presence of EGF. As in Figure 5, the model assumes the presence of scaffolding proteins KSR1 as outlined in the text. Top panel represents the physical characteristics of pRAF, pMEK and pERK both free and bound to KSR1 used in the computer simulations. Lower panels represent predicted steady state distribution of pRAF, pMEK, and pERK in normal cells assuming continuous presence of ligand at the cell membrane and assuming the presence of scaffolding protein KSR1. Middle panels are actual distribution observed in HMEK cells in culture using CYTOO chips so that every cell maintains the same shape. Arrows on the left middle panel denote the approximate location of the CM.

Changing the pH of the cytoplasm will change the distribution of both the unphosphorylated and phosphorylated messenger proteins by their isoelectric points. This phenomena can be compared to performing isoelectric focusing technique on IPG gels with different pH ranges. For example, if the cytosolic pH drops dramatically then a different set of proteins with lower pI’s will be separated out instead of the ones separated by a normal pH. This could be interesting to study what pH ranges of different pathways result in the most efficient information transfer for that pathway. For example, it is possible to negate the MAPK pathway dynamics if the pH drops below 6.0 in the cytoplasm. In this way the RAF, MEK, and ERK, would all be pushed to the cell wall, breaking down the organization and efficiency. This example seems extreme but it is easy to imagine that some pathways take a much less drastic change in pH to affect.

**Figure 7:**
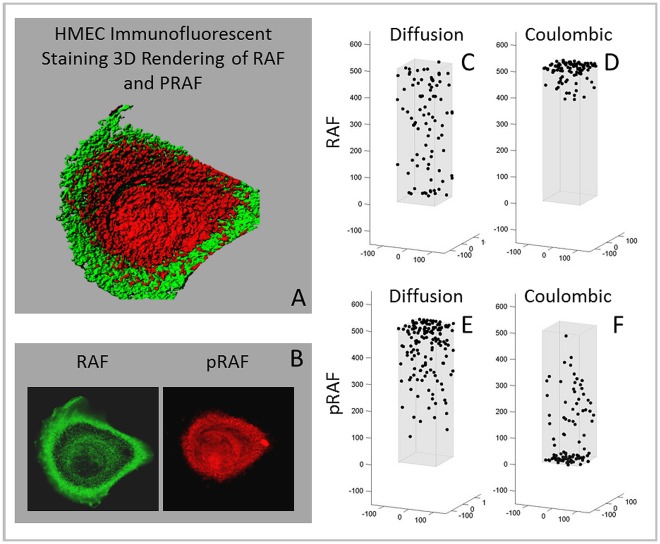
Predicted and measured steady state distribution of RAF and pRAF under typical culture condition with continuous ligand binding of EGFR on the cell membrane. The left panels demonstrate typical pRAF and RAF distribution in cultured HMEK cells. A) An iso-rendered distribution of RAF (green) and pRAF (red) with B) the photomicrograph demonstrating RAF clustered around the CM and pRAF clustered around the NM, as predicted by the IEM simulations. In the right panels, computer simulations demonstrate that if protein movement is by diffusion alone, pRAF will exhibit a concentration gradient with highest levels near the CM where it is generated by interactions with RAS and lowest levels near the nucleus. RAF will be widely dispersed. In the IEM simulations, pRAF will, due to its negative charge, concentrate near the nucleus while RAF (with an IEP of 9.2) will cluster near the CM.

When we assumed movement governed by only random walk, the distribution of pRAF, pMEK, and pERK reflected a concentration gradient from the CM which was the starting point in the cascade to the NM. The distribution of RAF, MEK and ERK represented a roughly opposite distribution. The expected distribution assume an intracellular field and pH gradient is shown in Figures 5 and 6. As in prior simulations, RAF holds a positive charge within the pH of the cytoplasm, and localized to the cytoplasm adjacent to the CM. Free MEK and ERK (isoelectric points of 6.1 and 6.2 respectively) localize adjacent to the nuclear membrane. However, when MEK and ERK that are bound to KSR1 (with an IEP of 9.1), we assume the complex to have an IEP that is the summation of the associate proteins. In this case, the MEK and ERK bound to KSR1 will tend to move peripherally away from the NM. pERK and pMEK tend to move rapidly to the NM and so high concentrations in the perinuclear cytoplasm were predicted (Figure 7). However, pMEK and pERK bound to KSR1 will tend to move somewhat into the cytoplasm due to the relatively high IEP of KSR1.

#### Experimental determination MAPKK proteins distribution and movement

The model simulations were then compared to experimental observations. Distribution RAF, pRAF, MEK, pMEK, ERK and pERK were determined in HMEC cells grown on CYTOO chips with a triangular micro pattern of fibronectin so that several thousand identically shaped and isolated cells were present on each chip. This allowed reliable measurement of protein distribution without the confounding effects of changes in cell morphology due to variability in binding or cellular crowding. Representative images of protein localization are shown in Figures 4 and 5. The experimental observations are virtually identical to those predicted by the computer simulations that assume the presence of a pH gradient and intracellular electric field. Unphosphorylated RAF was consistently demonstrated to cluster in the cytoplasm adjacent to the CM. By contrast, and again consistent with model predictions, pRAF was observed adjacent to the NM. Both phosphorylated and unphosphorylated MEK and ERK clustered near the NM as was expected from model simulations (Figure 7).

## Discussion

In the current model of cellular biology, protein interactions are usually depicted as a “hairball" diagram [19] which has no spatial explicitly component so that the intracellular localization and physical movement of protein is not considered. In general, macromolecules not localized within organelles are assumed to freely diffuse in a well-mixed cytosol. Localization and movements of messenger proteins that transduce signals from the CM to the N are similarly presumed governed by random walk. Figure 1 depicts this conventional view in which signal transduction by random walk appears straight-forward. However, this is not drawn to scale as the distance between the CM and the NM is typically about 1,000 protein diameters. Furthermore, while scaffolding proteins may promote interactions between the components of the pathway, they still must gain proximity through random impacts limiting the speed and efficiency of the process. Finally, the current models signal transduction does not permit communication of temporal and spatial information in ligand binding. However, such information appears important in cellular response to, for example, gradients of environmental signals [20]. Indeed, in developmental biology there is evidence that spatial information is obtained and used by cells through detection of gradients around their circumference [21], [22].

We propose intracellular protein localization and movement are also dependent on two components of the cellular physical microenvironment: a radially directed electric field from the NM into the CM and variations in cytoplasmic pH with increased acidity in the cytosol adjacent to the NM and the CM. The range of intracellular pH has been extensively studied using multiple techniques. Here, we used a fluorescence pH reporter (SNARF) to confirm a pH range of 7.4 to 7.2. The proposed intracellular electric field is proposed with limited prior theoretical and experimental investigation [1], [10] (Figure 1). Tyner et al. [10] clearly demonstrated the presence of an intracellular field although the source of the field remains unclear. We have speculated that it may be related to charges on the nuclear membrane that remain unshielded due to mobile ion flow through the nuclear pores. Colwell et al. [23] recently demonstrated that charges on proteins within the nuclear pore complex were critical for electrostatic interactions and that a net negative charge at pH 7.2 is necessary trait for translocation competent proteins. Furthermore, other authors have noted the role of electrostatic forces in actin polymerization [24], mitosis [25], and microtubules [26].

As depicted above, these previously unknown mechanisms provide a mechanism for controlling the optimal localization of messenger proteins within the cytoplasm as well rapid movement from the CM to the NM preserving information on the location and time of ligand binding. The predictions of the model are consistent with experimental observations in the MAPK pathway.

While we apply the model to messenger proteins, we note that the principles developed here should be general. That is, it is reasonable to assume that, for example, proteins involved in glucose metabolism that are not bound to the mitochondria will be organized in the cytosol based on their IEP. Furthermore, disruption of signaling pathways is nearly universally observed in cancers and so it is likely that variations in IEFM will be observed during carcinogenesis.

Finally, we note that there are no currently known mechanisms by which the nucleus can deconvolve and use spatial and temporal information regarding ligand binding once it arrives at the nucleus. Thus, it is an explicit prediction of this model that such mechanisms will be found within the compartmentalization of the nucleus.

## Supporting Information

Figure S1 **The deviation of the simulation RMS from the theoretical RMS at large ∂t values is due to the fact that the particles are confined to the inside of the cell wall.** This will cause the RMS to plateau instead of increasing along with the theoretical.
(DOC)

Figure S2 **Speciation diagram of a particle with an isoelectric point of 6.5.** These speciation are used to determine the charge of a particle at any given environmental pH.
(DOC)
