# Supplementary material for: Intracellular Electric Field and pH Optimize Protein Localization and Movement
Source: PLoS One. 2012 May 18;7(5):e36894. doi: 10.1371/journal.pone.0036894 (PMC3356409; doi:10.1371/journal.pone.0036894)
Supplement: Figure S1 — The deviation of the simulation RMS from the theoretical RMS at large ∂t values is due to the fact that the particles are confined to the inside of the cell wall. This will cause the RMS to plateau instead of increasing along with the theoretical. (DOC) [file pone.0036894.s001.doc]

Figure S1. The deviation of the simulation RMS from the theoretical RMS at large
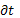
 values is due to the fact that the particles are confined to the inside of the cell wall. This will cause the RMS to plateau instead of increasing along with the theoretical.
