# Supplementary material for: Intracellular Electric Field and pH Optimize Protein Localization and Movement
Source: PLoS One. 2012 May 18;7(5):e36894. doi: 10.1371/journal.pone.0036894 (PMC3356409; doi:10.1371/journal.pone.0036894)
Supplement: Figure S2 — Speciation diagram of a particle with an isoelectric point of 6.5. These speciation are used to determine the charge of a particle at any given environmental pH. (DOC) [file pone.0036894.s002.doc]

Figure S2. Speciation diagram of a particle with an isoelectric point of 6.5. These speciation diagrams are used to determine the charge of a particle at any given environmental pH.
